# Enhancement of Dissipative Sensing in a Microresonator Using Multimode Input †

**DOI:** 10.3390/s22176613

**Published:** 2022-09-01

**Authors:** Sreekul Raj Rajagopal, A. T. Rosenberger

**Affiliations:** Department of Physics, Oklahoma State University, Stillwater, OK 74078, USA

**Keywords:** microresonator, whispering-gallery modes, dissipative sensing, multimode fiber

## Abstract

Optical whispering-gallery microresonators have proven to be especially useful as chemical sensors. Most applications involve dispersive sensing, such as the frequency shift of resonator modes in response to a change in the ambient index of refraction. However, the response to dissipative interaction can be even more sensitive than the dispersive response. Dissipative sensing is most often conducted via a change in the mode linewidth owing to absorption in the analyte, but the change in the throughput dip depth of a mode can provide better sensitivity. Dispersive sensing can be enhanced when the input to the microresonator consists of multiple fiber or waveguide modes. Here, we show that multimode input can enhance dip-depth dissipative sensing by an even greater factor. We demonstrate that the multimode-input response relative to single-mode-input response using the same fiber or waveguide can be enhanced by a factor of more than one thousand, independent of the mode linewidth, or quality factor (*Q*), of the mode. We also show that multimode input makes the dip-depth response nearly one hundred times more sensitive than the linewidth-change response. These enhancement factors are predicted by making only two measurements of dip depth in the absence of an analyte: one with the two input modes in phase with each other, and one with them out of phase.

## 1. Introduction

Optical microresonators have been a topic of much research and application in recent decades. They typically have high quality factors and small mode volumes and hence have been widely used as high-sensitivity sensors [1]. In particular, whispering-gallery mode (WGM) microresonators have been used to monitor changes in pressure, temperature, chemical composition, and refractive index, as well as other quantities [2,3].

A microresonator such as a sub-mm-diameter fused silica microsphere can support optical modes known as WGMs. In a WGM, light circulates by total internal reflection in the sphere’s equatorial plane, just under the surface, with a resonance when there are an integral number of wavelengths around the circumference. Light is coupled into a WGM from an optical fiber, tapered to a waist diameter of about 2 μm, tangent to the microsphere. The optical power transmitted down the fiber after the microsphere (throughput) is monitored as the frequency of the input light is scanned. Because of the microsphere’s intrinsic loss, *αL*, due to scattering and absorption, the throughput power shows a Lorentzian dip as the frequency scans through a WGM resonance. The light circulating in a WGM experiences another loss, *T*, denoting outcoupling back into the fiber waist. The linewidth of the Lorentzian dip is proportional to the total loss, *αL* + *T*, but the relative throughput dip depth *M* depends on the ratio of the losses, *y* = *αL*/*T*:

(1) $$M=\frac{4y}{{\left(1+y\right)}^{2}}.$$

*M* is the depth of the Lorentzian resonance dip relative to the throughput off resonance. Off resonance, no light is coupled into the WGM, and the throughput equals the input. Note that *M* << 1 for *y* << 1 (strong overcoupling) and for *y* >> 1 (strong undercoupling), but *M* = 1 for *y* = 1, leading to full extinction of the throughput. This condition, of *αL* = *T* and *M* = 1 (zero throughput), is referred to as critical coupling. Because light in the WGM is confined by total internal reflection, it has an evanescent component outside the microresonator that can interact with the environment, enabling dispersive and dissipative sensing of chemicals in the ambient medium.

For chemical sensing, optical WGM sensors detect via the registration of changes in their throughput spectral response due to perturbations in the environment probed by the evanescent (or interacting) fraction [4] of the WGM. The most-used spectral features of a WGM are its resonance frequency and linewidth, used for dispersive and dissipative sensing, respectively. The resonance frequency of a WGM shifts with a change in the refractive index, whereas loss mechanisms such as absorption and scattering in the ambient medium can increase the linewidth of a WGM. In addition to the change in linewidth, dissipative phenomena also induce a change in the resonant throughput dip depth, and thus dissipative sensing can be studied in detail by monitoring the change in the dip depth of a WGM.

Previously, it was demonstrated that absorption sensing based on fractional dip depth change could provide better sensitivity than frequency shift measurements [5]. Experimental confirmation [6] was provided by introducing trace gases into the surroundings of a cylindrical fused silica microresonator, and strain-tuning a WGM through a trace gas absorption line. The WGM’s effective intrinsic loss becomes modified and hence, on resonance with the gas absorption, the dip depth changes by an amount that depends on the external evanescent fraction of the WGM interacting with its surroundings. In addition to the external evanescent fraction, the internal evanescent (or interacting) fraction of a hollow microresonator, which can be much larger than the external fraction, can also be used for sensing purposes [2,3]. A thin-walled hollow bottle resonator (HBR) is ideally suited for use as a WGM-based optical absorption sensor [3,7,8]. More evidence for microresonator-based dissipative sensing being more sensitive than dispersive sensing has recently been provided [9,10,11,12,13,14].

In this work, we show how dissipative sensing in a microresonator can be enhanced by using multimode input. Light is coherently coupled into a single microresonator mode simultaneously from two waveguide or tapered-fiber modes, but only the throughput on the fundamental mode is detected. The interference effects involved in the addition of the outcoupled light to the “reflected” light (not coupled into the microresonator) to form the throughput can make the dip depth very sensitive to analyte absorption. We show that the sensitivity (fractional change in dip depth for a given analyte concentration) can be enhanced more than thousandfold compared to the sensitivity of the same system with single-mode input. We further show that the sensitivity can be nearly a hundred times that of using the change in WGM linewidth for detection of the same analyte. These enhancement factors are independent of system details and are predicted by making only two measurements of dip depth in the absence of analyte: one with the two input modes in phase with each other, and one with them out of phase. We show that this dissipative sensing technique can easily be implemented with a tapered-fiber-coupled HBR for internal detection of an analyte in solution or trace gas in air.

## 2. Materials and Methods

**Figure 1:**
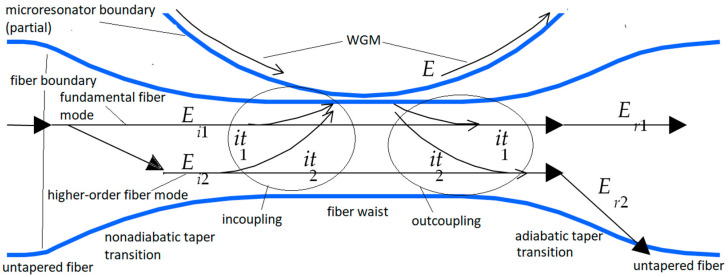
Multimode input to a microresonator. (Fiber not to scale but enlarged to show detail). Light in the untapered single-mode fiber strongly couples into two fiber waist modes of amplitudes *E_i_*_1_ and *E_i_*_2_. They then couple weakly into a WGM of amplitude *E* with input coupling coefficients *it*_1_ and *it*_2_, respectively. The WGM then couples out into throughput modes of amplitudes *E_r_*_1_ and *E_r_*_2_ with output coupling coefficients equal to the input coefficients. Only the fundamental throughput mode survives to be detected, since the higher-order mode cannot propagate in the untapered single-mode fiber.

The results presented in the next section are based on the following method of analysis of the system described in the last paragraph of the Introduction. First, consider the system in more detail. Typically, a WGM of a microresonator is excited by coupling in tunable laser light from a single fiber mode, using an adiabatically tapered fiber. The enhancement scheme uses a non-adiabatic tapered fiber to couple light from two fiber modes into the microresonator, as illustrated in Figure 1. The WGM will belong to one of two polarization families, TE (transverse electric) or TM (transverse magnetic). The input light in the fiber will be linearly polarized to match the WGM polarization. 

The situation illustrated in Figure 1 shows a single-mode fiber with an asymmetric bitaper producing a microfiber waist. Because the downtaper is nonadiabatic, two modes are excited on the waist: fundamental (1) and higher-order (2); they couple into the WGM. Outcoupled light goes into both waist modes, but only the fundamental survives passage through the adiabatic uptaper. The resonant throughput dip is monitored for enhanced change due to absorption in an analyte interacting with the WGM’s evanescent fraction. This technique is generic; the specifics of the waveguide and microresonator do not matter. What is needed is two-mode input, with the two input (waist) modes having different propagation constants so that their relative phase on coupling into the microresonator depends on the position of the coupling point along the fiber waist; a filter (adiabatic uptaper) to ensure that throughput on only one mode (the fundamental) is detected; and a dissipative loss that changes the net intrinsic loss of the microresonator’s mode (such as an absorbing analyte interacting with a WGM’s evanescent fraction). The theory presented here thus applies to a large possible range of waveguide–resonator systems. A brief sketch of this theory [15] and preliminary experimental confirmation [16] have been presented earlier, and full experimental results will be published elsewhere [17].

The coupling of incident tapered fiber modes into the WGM is coherent, that is, the amplitude coupled into the WGM is the sum of the complex amplitudes of the coupled portions of the two incident modes. The independence of the waist modes, and the condition that energy be conserved when the cavity has no intrinsic loss, lead to certain relations among the coupling (transmission) and reflection coefficients. If

(2) $$\begin{array}{l} t_{1}^{2}=1-r_{1}^{2},\\ t_{2}^{2}=1-r_{2}^{2}, \end{array}$$

where *it_n_* and *r_n_* are the coupling and external reflection coefficients for mode *n*, energy conservation requires that

(3) $$1-r^{2}=t_{1}^{2}+t_{2}^{2}=T_{1}+T_{2},$$

where *r* is the internal reflection coefficient for the cavity mode and *T_n_* is the transmissivity for mode *n*. The transmissivities are assumed to be small, i.e., *T_n_* << 1.

Consider cw incident waves and steady-state response. *E_i_*_1_ and *E_i_*_2_e*^iβ^* are the amplitudes of the two incident modes, with their relative phase *β* depending on the position of the microresonator along the fiber waist. Then, the intracavity WGM mode amplitude *E*, just after the input coupling, is given by

(4) $$E=\frac{it_{1}E_{i1}+it_{2}E_{i2}e^{i\beta }}{1-re^{-\alpha L/2}e^{i\delta }},$$

where *L* is the cavity round-trip length, *αL* is the intrinsic round-trip power loss, and *δ* is the round-trip phase accumulation modulo 2*π*, proportional to the detuning of the incident light from the cavity resonance. Since we assume that no intermode coupling occurs in the second transition region (adiabatic uptaper), and that mode 2 is lost while mode 1 is captured by the untapered fiber core, the throughput amplitude of mode 1 becomes

(5) $$E_{r1}=r_{1}E_{i1}+it_{1}Ee^{-\alpha L/2}e^{i\delta }=r_{1}E_{i1}-\frac{t_{1}^{2}E_{i1}+t_{1}t_{2}E_{i2}e^{i\beta }}{1-re^{-\alpha L/2}e^{i\delta }}e^{-\alpha L/2}e^{i\delta }.$$

However, power is what we measure, so we want the square modulus of this. We can evaluate to the lowest order in the small quantities *T_n_* << 1, *αL* << 1, *δ* << 1. The last condition applies because *δ* increases by 2π when the detuning equals the free spectral range of the cavity, the cavity *Q* (and thus its finesse) is very high, and we must be relatively near resonance (within several linewidths). Then we find

(6) $${\left|E_{r1}\right|}^{2}={\left|\frac{\left(\frac{T_{2}+\alpha L-T_{1}}{2}-i\delta \right)E_{i1}-\sqrt{T_{1}T_{2}}E_{i2}e^{i\beta }}{\frac{T_{1}+T_{2}+\alpha L}{2}-i\delta }\right|}^{2}.$$

Far away from the cavity resonance, at large detunings, *δ*, note that the limiting value of ${\left|E_{r1}\right|}^{2}$ is ${\left|E_{i1}\right|}^{2}$. It is therefore convenient to describe the resonance throughput dip (or peak, or feature, in general) in terms of a relative throughput power *R*; note that a peak means that *R* > 1. With the definition of *m* = *E_i_*_2_/*E_i_*_1_, we have:

(7) $$R_{\delta \beta }={\left|\frac{E_{r1}}{E_{i1}}\right|}^{2}={\left|\frac{\left(\frac{T_{2}+\alpha L-T_{1}}{2}-i\delta \right)-\sqrt{T_{1}T_{2}}me^{i\beta }}{\frac{T_{1}+T_{2}+\alpha L}{2}-i\delta }\right|}^{2}.$$

Equation (7) shows how the throughput spectrum can exhibit different features. For example, for *β* = 0 a symmetric dip is normally observed; for *β* = *π* a symmetric peak can appear; and for arbitrary *β* an asymmetric Fano-like lineshape will result. The multimode input can make this Fano lineshape steeper than normal, resulting in enhanced dispersive sensitivity [18,19,20,21,22,23]. We show here that even greater enhancement can be produced for the dissipative sensitivity. To that end, note that on resonance (*δ* = 0) Equation (7) can be written as

(8) $$R_{0\beta }=\frac{{\left(T_{1}-T_{2}-\alpha L\right)}^{2}+4T_{1}T_{2}m^{2}+4\left(T_{1}-T_{2}-\alpha L\right)\sqrt{T_{1}T_{2}}m\;\cos\beta }{{\left(T_{1}+T_{2}+\alpha L\;\right)}^{2}}.$$

In the next section we show how application of the method detailed above leads to the sensitivity enhancements described in the Introduction. All the assumptions made in this section are experimentally realistic, with one exception: the input and output coupling coefficients may not be equal, because phase matching is not required for efficient fiber–resonator coupling. The assumption of equality is often reasonable [24] but will not hold in general [25]. However, as we show below, whether the input and output coupling coefficients are equal is irrelevant to our results.

## 3. Results

### 3.1. Enhancement—Two-Mode vs. One-Mode

Note that the relative throughput power in Equation (8) depends on the three quantities *T*_1_, *T*_2_ + *αL*, and *T*_2_*m*^2^. Since the total loss is related to the linewidth Δ*ν* of the mode by

(9) $$T_{1}+T_{2}+\alpha L=\frac{4\pi^{2}na\Delta \upsilon }{c},$$

where *a* is the microresonator radius, *n* is the WGM’s effective refractive index, and *c* is the speed of light, the values of the three quantities can be found by measuring the linewidth along with *R*_00_ and *R*_0*π*_; details will be given in a later subsection. The dip depth is given by

(10) $$M_{0\beta }=1-R_{0\beta }=\frac{4T_{1}\left(T_{2}+\alpha L\right)-4T_{1}T_{2}m^{2}-4\left(T_{1}-T_{2}-\alpha L\right)\sqrt{T_{1}T_{2}}m\;\cos\beta }{{\left(T_{1}+T_{2}+\alpha L\right)}^{2}}.$$

Our dissipative sensing is based on measuring the fractional change in dip depth (with *β* = 0) due to absorption by the analyte effectively increasing the intrinsic loss by *dαL*. The effective intrinsic loss can be written as

(11) $$\alpha L=\alpha_{i}L+f\alpha_{s}L+f\alpha_{a}L,$$

where *α_i_* is the intrinsic loss coefficient of the microresonator which includes scattering, absorption, and radiation losses, *α_s_* is the absorption coefficient of the solvent (if necessary), *α_a_* is the absorption coefficient of the analyte, and *f* is the fraction of the WGM [4] which interacts with the solvent and analyte: thus, *dαL* = *fdα_a_L*. The fractional change in dip depth is expressed in terms of the derivative of *M*_00_ with respect to *αL*:

(12) $$\frac{1}{M_{00}}\frac{dM_{00}}{d\alpha L}=\frac{T_{1}\left(T_{1}-T_{2}-\alpha L\right)+2T_{1}T_{2}m^{2}+\left(3T_{1}-T_{2}-\alpha L\right)\sqrt{T_{1}T_{2}}m}{\left(T_{1}+T_{2}+\alpha L\;\right)\left\{T_{1}\left(T_{2}+\alpha L\right)-T_{1}T_{2}m^{2}-\left(T_{1}-T_{2}-\alpha L\right)\sqrt{T_{1}T_{2}}m\right\}}.$$

If *m* = 0, Equation (12) gives the fractional change in dip depth for the same additional loss that would be found using the same waveguide or microfiber waist, but with only the fundamental mode incident. The absolute value of the ratio of Equation (12) with arbitrary *m* to Equation (12) with *m* = 0 thus gives the sensitivity enhancement factor of two-mode input relative to one-mode input:

(13) $$\eta_{21}=\left|\frac{\left(T_{2}+\alpha L\right)\left[T_{1}\left(T_{1}-T_{2}-\alpha L\right)+2T_{1}T_{2}m^{2}+\left(3T_{1}-T_{2}-\alpha L\right)\sqrt{T_{1}T_{2}}m\right]}{\left(T_{1}-T_{2}-\alpha L\;\right)\left\{T_{1}\left(T_{2}+\alpha L\right)-T_{1}T_{2}m^{2}-\left(T_{1}-T_{2}-\alpha L\right)\sqrt{T_{1}T_{2}}m\right\}}\right|.$$

**Figure 2:**
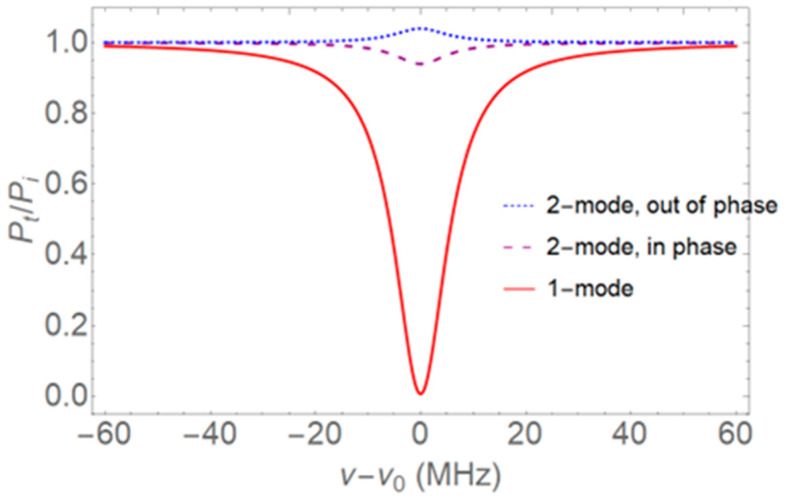
Conditions leading to large sensitivity enhancement. Relative throughput power *R* = *P_t_*/*P_i_* from a fiber-coupled microresonator WGM is plotted as a function of detuning from WGM resonance. With two incident fiber modes, a shallow dip results if they are in phase (purple dashed curve, *R*_00_ = 0.940) and a small peak appears if they are out of phase (blue dotted curve, *R*_0*π*_ = 1.040). With one incident fiber mode, the coupling is near critical (red solid curve, *M* = 0.9937).

We see that the enhancement depends on the same three quantities as the relative throughput power in Equation (8), *T*_1_, *T*_2_ + *αL*, and *T*_2_*m*^2^; if they are all nearly equal, the denominator in Equation (13) becomes small and the enhancement will be large. For example, if *T*_2_*m*^2^ is just slightly larger than *T*_1_, and *T*_2_ + *αL* is just slightly larger than *T*_2_*m*^2^, it can be seen from Equation (8) that the throughput for *β* = 0 will show a shallow dip (*R*_00_ < 1) and the throughput for *β* = *π* will have a small peak (*R*_0*π*_ > 1). For *m* = 0, Equation (8) shows near critical coupling (*R* ~ 0). An example of the observations that indicate the near equality of *T*_1_, *T*_2_ + *αL*, and *T*_2_*m*^2^, and thus a large enhancement factor, are illustrated in Figure 2. Assume a WGM with a linewidth of 12 MHz (*Q* = 1.61 × 10^7^ for a wavelength of 1550 nm), where it is found that *R*_00_ = 0.940 and *R*_0*π*_ = 1.040 for two-mode input, and where one-mode input results in near-critical coupling with *M* = 0.9937. These are conditions that are easily achievable experimentally.

Observations of throughput spectra like those shown in Figure 2 predict a large enhancement factor. Before analyzing what is meant by this large sensitivity enhancement, consider some of the assumptions made. It is assumed that there are two incident modes; however, the microfiber waist has many modes that are above cutoff. Nevertheless, with a proper design of the non-adiabatic downtaper it is possible to excite only the fundamental and one higher-order mode (or at least one cluster of higher-order modes with nearly equal propagation constants) [16,17]. It is further assumed that light in the microresonator couples out into only those two modes. Again, by choosing the ratio of the resonator radius to the fiber waist radius properly, it is possible to achieve this [24]. If there is some outcoupling into even higher-order modes, it will simply appear to be an extra intrinsic loss. By treating the change in dip depth as a differential, we seem to be implying that *dM*_00_/*M*_00_ << 1; however, *dM*_00_ = −*dR*_00_, and *R*_00_ is near 1, whereas |*M*_00_| << 1. The fractional change in throughput power *R*_00_ is small, so the differential formalism works even when *dM*_00_/*M*_00_ ~ 1. The remaining assumption is that the coupling coefficients for waist mode to resonator mode and resonator mode to waist mode are equal. In the next subsection, this assumption will be dropped, and we will see that our results are not affected.

### 3.2. Case of Different Input and Output Coupling

Revisit Figure 1, and now let the input coupling coefficients be *it_n_*, as before, but now call the output coupling coefficients *iτ_n_*. Output coupling is a loss for the resonator mode, so the linewidth gives the total loss again via the relation

(14) $$\tau_{1}^{2}+\tau_{2}^{2}+\alpha L=\frac{4\pi^{2}na\Delta \upsilon }{c}.$$

Now measuring *R*_00_ and *R*_0*π*_ allows the determination of two other quantities, 2*τ*_1_*t*_1_ and *τ*_1_*t*_2_*m*; along with the total loss (note that in the case of equal input and output coupling, the three quantities could have been chosen to be *T*_1_, *T*_1_ + *T*_2_ + *αL*, and *T*_2_*m*^2^), these three quantities are all that are needed to determine the enhancement, *η*_21_. Finding these three quantities using the same values of the linewidth, *R*_00_, and *R*_0*π*_ as in the previous subsection, we obtain the same value of *η*_21_.

This result is indicative of a deeper meaning. Because the cases of arbitrary *m* and *m* = 0 involve the same resonator mode and the same input/output coupling coefficients, any effect of input–output coupling difference cancels. This suggests that even further simplification is possible; note that the total loss does not depend on *m*, so the enhancement factor *η*_21_ turns out to be independent of the resonator mode linewidth or quality factor *Q*. Therefore, the enhancement can be found simply from the values of *R*_00_ and *R*_0*π*_; the situation where *R*_00_ is slightly less than 1 and *R*_0*π*_ is slightly greater than 1 will allow for large enhancement, as we now demonstrate explicitly.

### 3.3. Simple Expression for Enhancement Factor

To find the expression for the enhancement factor in terms of *R*_00_ and *R*_0*π*_, consider Equation (8) for the relative throughput power evaluated for the incident fiber modes in phase (*β* = 0) and out of phase (*β* = *π*):

(15) $$\begin{array}{l} R_{00}={\left(\frac{T_{1}-T_{2}-\alpha L+2\sqrt{T_{1}T_{2}}m}{T_{1}+T_{2}+\alpha L}\right)}^{2},\\ R_{0\pi }={\left(\frac{T_{1}-T_{2}-\alpha L-2\sqrt{T_{1}T_{2}}m}{T_{1}+T_{2}+\alpha L}\right)}^{2}. \end{array}$$

Then, with the following definitions,

(16) $$R_{1}=\sqrt{R_{0\pi }}-\sqrt{R_{00}}\;\mathrm{and}\;R_{2}=\sqrt{R_{0\pi }}+\sqrt{R_{00}},$$

we find that the three quantities of interest can be written as

(17) $$\begin{array}{l} T_{1}=\frac{\left(T_{1}+T_{2}+\alpha L\right)}{2}\left[1-\frac{1}{2}R_{1}\right],\\ T_{2}+\alpha L=\frac{\left(T_{1}+T_{2}+\alpha L\right)}{2}\left[1+\frac{1}{2}R_{1}\right],\\ 2\sqrt{T_{1}T_{2}}m=\frac{\left(T_{1}+T_{2}+\alpha L\right)R_{2}}{2}. \end{array}$$

Now notice that Equations (12) and (13) share a common factor; using Equations (16) and (17) to evaluate that factor in terms of *R*_00_ and *R*_0*π*_, Equations (12) and (13) become

(18) $$\frac{1}{M_{00}}\frac{dM_{00}}{d\alpha L}=\frac{1}{T_{1}+T_{2}+\alpha L}\left[\frac{2\sqrt{R_{00}}}{1-\sqrt{R_{00}}}\right]$$

and

(19) $$\eta_{21}=\left|\frac{T_{2}+\alpha L}{T_{1}-T_{2}-\alpha L}\right|\frac{2\sqrt{R_{00}}}{1-\sqrt{R_{00}}}=\left|\frac{1-\frac{1}{2}\left(\sqrt{R_{00}}-\sqrt{R_{0\pi }}\right)}{\sqrt{R_{00}}-\sqrt{R_{0\pi }}}\right|\frac{2\sqrt{R_{00}}}{1-\sqrt{R_{00}}}.$$

Now, since *R*_00_ is typically slightly less than 1 and *R*_0*π*_ is typically slightly greater than 1, Equation (19) can be approximated:

(20) $$\eta_{21}=\left|\frac{1-\frac{1}{2}\left(\sqrt{R_{00}}-\sqrt{R_{0\pi }}\right)}{\sqrt{R_{00}}-\sqrt{R_{0\pi }}}\right|\frac{2\sqrt{R_{00}}}{1-\sqrt{R_{00}}}\approx \left|\frac{2}{R_{00}-R_{0\pi }}\right|\frac{4}{M_{00}}.$$

Although the derivation of this simplified form of the enhancement factor *η*_21_ was conducted assuming equal input and output coupling coefficients, the same expression is found if this assumption is not made, as in the previous subsection.

Consider, for example, the conditions illustrated in Figure 2, where *R*_00_ = 0.940 and *R*_0*π*_ = 1.040. From Equations (16) and (17) it is seen that *T*_1_, *T*_2_ + *αL*, and *T*_2_*m*^2^ are all nearly equal; the exact expression in Equation (20) gives *η*_21_ = 1299 and the approximate expression gives *η*_21_ = 1333.

Thus, the sensitivity (fractional change in dip depth) enhancement for two-mode input compared to one-mode input can be greater than three orders of magnitude. Now consider the second factor in the expression for *η*_21_ in Equation (20). 

### 3.4. Enhancement—Dip-Depth vs. Linewidth

That second factor was seen in Equation (18) as well: it turns out to be the enhancement for dip-depth sensing relative to linewidth sensing, *η_dl_*. Consider the expression for the WGM linewidth in Equation (9). It is easy to see that

(21) $$\frac{1}{\Delta \nu }\frac{d\Delta \nu }{d\alpha L}=\frac{1}{T_{1}+T_{2}+\alpha L}.$$

Therefore, from Equation (18) we have

(22) $$\eta_{dl}=\frac{1}{M_{00}}\frac{dM_{00}}{d\alpha L}/\frac{1}{\Delta \nu }\frac{d\Delta \nu }{d\alpha L}=\frac{2\sqrt{R_{00}}}{1-\sqrt{R_{00}}}\approx \frac{4}{M_{00}}.$$

For *R*_00_ = 0.940, as in Figure 2, the exact value of *η_dl_* is 63.7 and its approximate value is 66.7. Thus, the dip-depth sensitivity is nearly two orders of magnitude greater than the linewidth sensitivity. Note especially that this enhancement factor, *η_dl_*, is independent of the WGM’s linewidth and *Q*; a larger *Q* will increase the dip-depth sensitivity by the same factor as that by which it increases the linewidth sensitivity.

A few other enhancement factors, for experimental values of *R*_00_ and *R*_0*π*_, are shown in Table 1. Both the enhancement of two-mode input relative to one-mode input, *η*_21_, and the enhancement of dip-depth sensing relative to linewidth sensing, *η_dl_*, are given, showing that the conditions of Figure 2 are typical.

## 4. Discussion

The origin of the large sensitivity enhancement factor for two-mode input compared to one-mode input can be understood in terms of the coherent coupling of the fiber modes into and out of the WGM. Consider one-mode input: the near equality of *T*_1_ and *T*_2_ + *αL* means that the coupling is near critical, as illustrated by the deep dip in Figure 2. Under these conditions the outcoupled part of the intracavity field, which is out of phase with the input field because of the product of the input coupling coefficient *it*_1_ (*it*_1_ or *it*_2_ in the two-mode case) and output coupling coefficient *it*_1_, nearly cancels the uncoupled input field, so there is very little throughput power. On the other hand, when there is two-mode input with the two incident modes in phase and *T*_1_ and *T*_2_*m*^2^ nearly equal, the intracavity field will be roughly twice as large, so the portion outcoupled into the fundamental fiber mode will be nearly twice as great as the uncoupled input field, but out of phase with it, resulting in only a small observed change in throughput power (shallow dip). Absorption by the analyte reduces the very large intracavity field by a small fraction, but that then translates to a substantial change in the throughput dip depth when the outcoupled field interferes with the uncoupled fundamental input field.

The very large value of the enhancement factor *η*_21_ is therefore due, in part, to the fact that the coupling is near critical in the one-mode case, where the dip-depth sensitivity is expected to be low [5]. However, the two-mode dip-depth sensitivity is even greater than that of the one-mode case far from critical coupling, as seen by the following. Since *Q* = *ν*/Δ*ν*, Equation (9) shows that the first factor on the right-hand side of Equation (18) is proportional to *Q*. Now evaluate the right-hand side of Equation (12) with *T*_2_ = 0, as in the ideal one-mode case, and note that it is proportional to the intrinsic quality factor *Q_i_*, which is inversely proportional to *αL*. Equating the left-hand sides of these two equations (the dip-depth sensitivity) results in the following relation (*x* = *T*_1_/*αL*, and can range from <<1 to >>1):

(23) $$Q_{i}=\left|\frac{1+x}{1-x}\right|\frac{2\sqrt{R_{00}}}{1-\sqrt{R_{00}}}Q\approx \left|\frac{1+x}{1-x}\right|\frac{4}{M_{00}}Q.$$

This says that to have the same dip-depth sensitivity with a one-mode system, the resonator’s intrinsic quality factor must be *at least* a factor of 63.7 times as large as the *Q* in the two-mode system of Figure 2. This means *Q_i_* ~ 10^9^, which is pushing the limit of what can be achieved in fused silica.

Probably the most important result of this work is that the two-mode dip-depth sensitivity is quite a bit greater (nearly two orders of magnitude) than the sensitivity that would be found by measuring the relative change in WGM linewidth induced by the analyte absorption. This comparison is reminiscent of that of reference [26], where resonance amplitude is shown to be as important as the *Q* value for dispersive sensing in microresonators. In addition, because the experimental uncertainty in measuring dip depth is about 2%, compared to a 5% uncertainty in measuring linewidth, the dip-depth limit of detection (LOD) is enhanced by an extra factor of about 2.5 above the sensitivity enhancement *η_dl_*. For example, with the conditions of Figure 2, the dip-depth LOD would be about 160 times smaller than the linewidth LOD.

Dip-depth sensing has the advantages of being affected very little by drifts or fluctuations in input power or resonance frequency. Since the dip depth is a relative measure, it is independent of the input power. In addition, because the dip depth is measured at resonance, it is unaffected by a change in the resonance frequency.

As illustrated in Table 1, the enhancement factors found from the example of Figure 2, *η*_21_ = 1299 for two-mode vs. one-mode and *η_dl_* = 63.7 for dip-depth vs. linewidth, are typical and not overly optimistic estimates. The publication of experimental results for sensing of a dye in methanol solution, now in preparation [17], will show some cases with even greater enhancement factors than those of Figure 2. Note that our enhancement factors assume the same change in loss, *dαL* (see Equation (22), for example). Since *dαL* = *fdα_a_L*, to compare the responses for the same change in analyte absorption *dα_a_L*, *f* must have a fixed value; in other words, the same WGM must be used. In addition, since the fractional change in dip depth, *dM*_00_/*M*_00_, is proportional to *dαL*, the response to a given change in analyte absorption will be greater in the case of a larger interacting fraction, *f*, hence the advantage of internal sensing in a hollow resonator. It is our hope that this sensing enhancement will make near-IR detection of greenhouse gases such as methane and carbon dioxide in a hollow-bottle microresonator more feasible and help to avoid the need for complex and expensive mid-IR systems.

## Figures and Tables

**Table 1:** Enhancement factors. Two-mode input relative to single-mode input, *η*_21_, and dip-depth sensing relative to linewidth sensing, *η_dl_*.

*R*_00_	*R*_0*π*_	*η*_21_	*η_dl_*
0.940	1.040	1299	63.7
0.973	1.026	5542	145
0.911	1.043	648	41.9
0.936	1.040	1167	59.5

## Data Availability

Not applicable.
